# A pragmatic approach to estimating the cost to deliver and participate in implementation strategies

**DOI:** 10.1186/s13012-025-01459-y

**Published:** 2025-10-17

**Authors:** Hannah Cheng, Maryam Abdel Magid, Mark P. McGovern, James H. Ford, Veena Manja, Hélène Chokron Garneau, Todd H. Wagner

**Affiliations:** 1 Stanford Center for Dissemination and Implementation, Department of Psychiatry & Behavioral Sciences, Stanford University School of Medicine, 1070 Arastradero Road, Palo Alto, CA 94304-5590 USA; 2 Department of Medical Social Sciences, Feinberg School of Medicine, Northwestern University, Chicago, IL USA; 3 Department of Biomedical Informatics and Medical Education, UW Medicine, University of Washington, Seattle, WA USA; 4 Division of Primary Care and Population Health, Department of Medicine, Stanford University School of Medicine, Stanford, CA USA; 5 School of Pharmacy, Social and Administrative Sciences Division, University of Wisconsin, Madison, WI USA; 6 School of Medicine, University of California Davis, Sacramento, CA USA; 7 VA Northern California Health Care System, Mather, CA USA; 8 Stanford Surgery Policy Improvement and Education Center, Stanford Medicine, Stanford, CA USA; 9 Health Economics Resource Center, VA Palo Alto Health Care System, Menlo Park, CA USA

**Keywords:** Implementation cost, Economic evaluation, Micro-costing, Implementation strategies, Opioid use disorder

## Abstract

**Background:**

Implementation costs—the combined costs of *delivering* expert support and *participating* in an implementation endeavor—are often omitted from economic evaluations. When included, delivery and participation costs are usually combined, even though these may be covered by different funders. We propose a pragmatic micro-costing approach that separates the *delivery* and *participation* costs as well as outlines practical considerations for measuring implementation costs.

**Methods:**

Sixty-four specialty addiction treatment programs and primary care clinics participated in a stepped sequence of implementation strategies focused on improving access to buprenorphine and naltrexone for persons with opioid use disorder. The implementation strategies deployed were: audit and feedback (A&F), a two-day workshop, internal facilitation, and external facilitation. Our micro-costing approach separately measured the cost to *deliver* and *participate* in implementation strategies, as demonstrated through the A&F case example, which was the first of four implementation strategies deployed. We applied the following practical considerations to maximize the precision and accuracy of cost data: 1) Balance the frequency and length of cost survey, 2) Cost tracking training, 3) Regular survey reminders, 4) Tailor cost surveys, 5) Perform frequent cost data validation, 6) Iterative evaluation and refinement.

**Results:**

In A&F, the implementation setup cost was $32,266, and the annual recurring costs were $4,231 per clinic. While the majority of the setup cost (99%) can be attributed to A&F delivery, over half of the annual recurring costs (63%) were attributed to clinic participation in A&F.

**Conclusions:**

This micro-costing approach appears both pragmatic and meaningful. By understanding the total cost implications of implementation, decision-makers can better select the most suitable strategy based on the context, goals, and budget constraints to efficiently optimize the pace and desired outcome of an implementation endeavor.

**Trial registration:**

The trial protocol is registered with ClinicalTrials.gov (NCT05343793).

**Supplementary Information:**

The online version contains supplementary material available at 10.1186/s13012-025-01459-y.

Contributions to literature

- This pragmatic micro-costing approach serves as an easy-to-follow guide for capturing delivery and participation costs of implementation strategies across research and practice settings.
- We highlight practical considerations that balance feasible data collection while maximizing the accuracy and precision of cost data.
- Separately estimating delivery and participation costs can inform system leaders to select the most suitable strategy based on context, goals, and budget, especially when these costs are incurred in different budgets.

## Introduction

Cost information can help a wide range of healthcare decision-makers, improvement specialists, and researchers compare the economic ramifications of implementation strategies, the ‘how to” that promote the uptake of innovations in practice [1, 2]. Cost information also provides system leaders with concrete information that can aid in identifying the most appropriate strategy to utilize for the implementation of an innovation [3]. Providing transparency around implementation costs helps to guide decisions around whether an implementation is worth replicating given the budget constraints [4].

Implementation cost refers to “resources used to develop, execute, and partake in an implementation strategy” [1, 5]. As an example, Audit and Feedback (A&F) is a commonly used, evidence-based implementation strategy, where data are collected and fed back to implementation sites in dashboards to help them identify their strengths and opportunities for improvement [6, 7]. In this example, there are two streams of effort which incur costs. One is the *delivery* costs, which includes developing the dashboard, any technology, and the effort to provide the data back to the implementation sites. Put simply, this is the effort required to create the audit dashboards or reports and then share or feed them back to the sites. The second is the *participation* costs. This includes the time for clinic staff to review the dashboard, identify improvements, and set goals.

While health economists and implementation scientists have developed methods for estimating implementation costs, few studies to date have separately measured the cost to deliver and participate in implementation strategies [8, 9]. Many studies have estimated the cost to participate in implementation strategies without also considering the cost to deliver them [10, 11], or they combine them assuming that the “payer” is covering both costs. However, it is not uncommon, especially in mental health and substance use treatment, for different funders to separately finance these efforts. Block grants as well as direct financing have been used by states, Health Resources and Services Administration (HRSA), and Substance Abuse and Mental Health Services Administration (SAMHSA) to pay for delivering implementation efforts. If the delivery costs are financed separately, then not measuring delivery cost underestimates the total implementation cost, which can be particularly harmful in low-resourced settings given limited budget and human capital [12].

Among prior studies that have examined implementation cost, data collection methods varied in efforts to reduce the burden of collecting activity data from which costs can be estimated [13]. While several implementation cost estimation tools and frameworks are available [14–16], there are few pragmatic guides on how to do this in a large-scale implementation trial where separating delivery costs from participation costs is needed [11]. Implementation scientists and public health practitioners need simple and pragmatic methods when estimating implementation costs [13].

This manuscript introduces a micro-costing approach, the DISCo (Delivering Implementation Strategy Cost), to capture the costs of delivering implementation strategies. The DISCo builds on existing methods to separate the cost to deliver and participate in implementation strategies [14–16]. To illustrate its use, the DISCo is applied in A&F within an adaptive implementation trial designed to expand access to medications for opioid use disorder (MOUD) [3]. We also describe practical considerations to optimize the precision and accuracy of cost data. The micro-costing approach presented here can serve as a practical guide for implementation scientists or practitioners, whether in research settings or practice (e.g., health care organizations, schools, community centers), to optimize implementation cost estimation. Understanding the costs of implementing an innovation can help bridge the research-to-practice gap [17], ultimately improving access to high quality health care.

## Methods

### Study overview

**Fig. 1:**
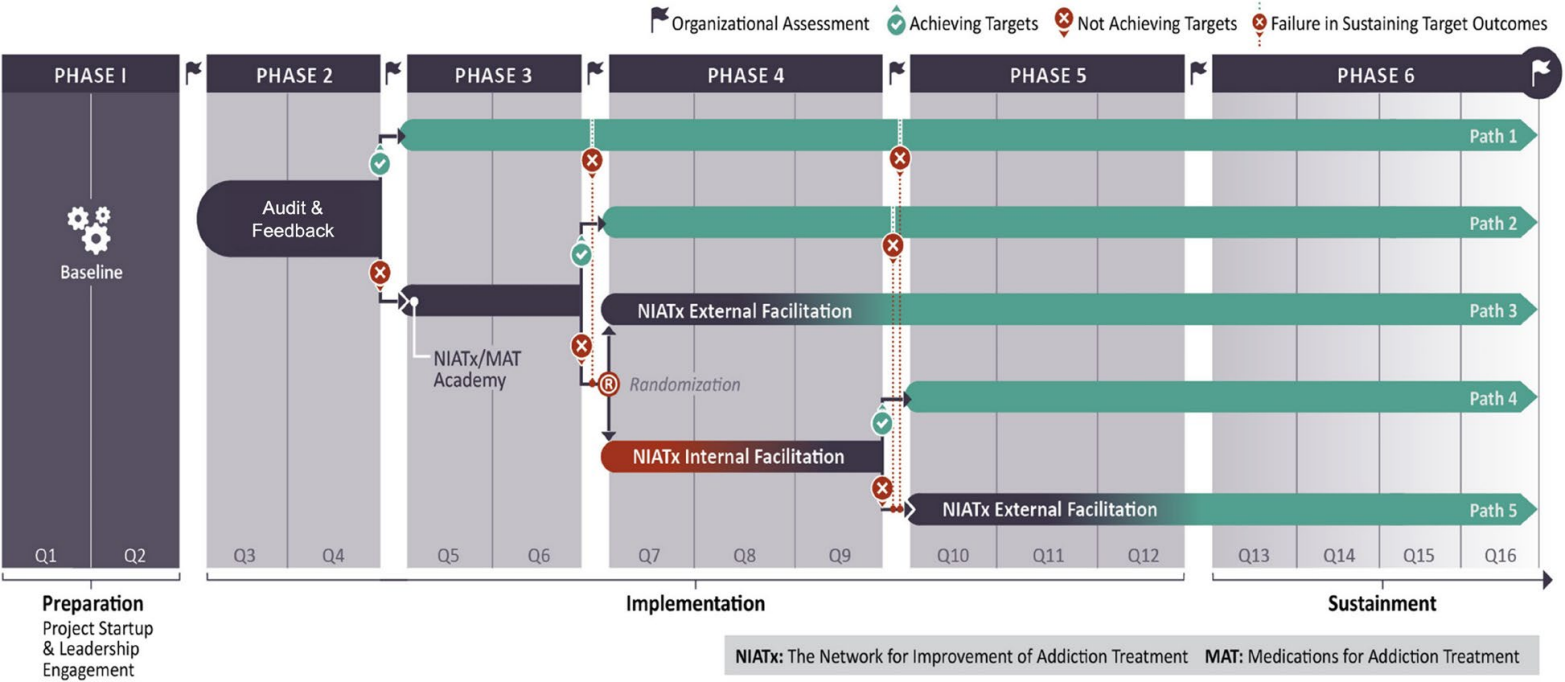
SITT-MAT adaptative implementation strategy design

The Stagewise Implementation-To-Target – Medications for Addiction Treatment (SITT-MAT) is an adaptive implementation trial designed to start up or scale up access to MOUD. Participants were 64 addiction specialty treatment programs and primary care clinics in a western US state. Clinics were offered an escalating intensity sequence of strategies in a stepwise fashion—Audit and Feedback (A&F), Two-Day Workshop, Internal Facilitation, and External Facilitation—based on whether they have met pre-determined target criteria at each step (Fig. 1). The trial is registered with ClinicalTrials.gov (NCT05343793) and the full study protocol is described [3]. Ethics approval was granted by the Stanford Institutional Research Board (IRB-66398).

The SITT-MAT economic evaluation aim was to prospectively estimate implementation costs from the implementation strategy actor (deliverer) and target recipient (participant) perspectives. We measured the costs associated with the delivery of and participation in the sequence of implementation strategies, analogous to the actor and target as described by Proctor [18]. The unit of analysis was the clinic. The time horizon was 36 months: *Pre-implementation* includes 6 months of preparation; *Active implementation* includes 6 months of active A&F, 6 months of two-day workshop, 9 months of randomization to either internal or external facilitation, and finally, 9 months of external facilitation only. This economic evaluation aligns with the guidance on the Consolidated Health Economic Evaluation Reporting Standards (CHEERS) [19] checklist (Supplementary File 1).

### Micro-costing procedures

#### Step 1: Determine what cost should be measured

**Fig. 2:**
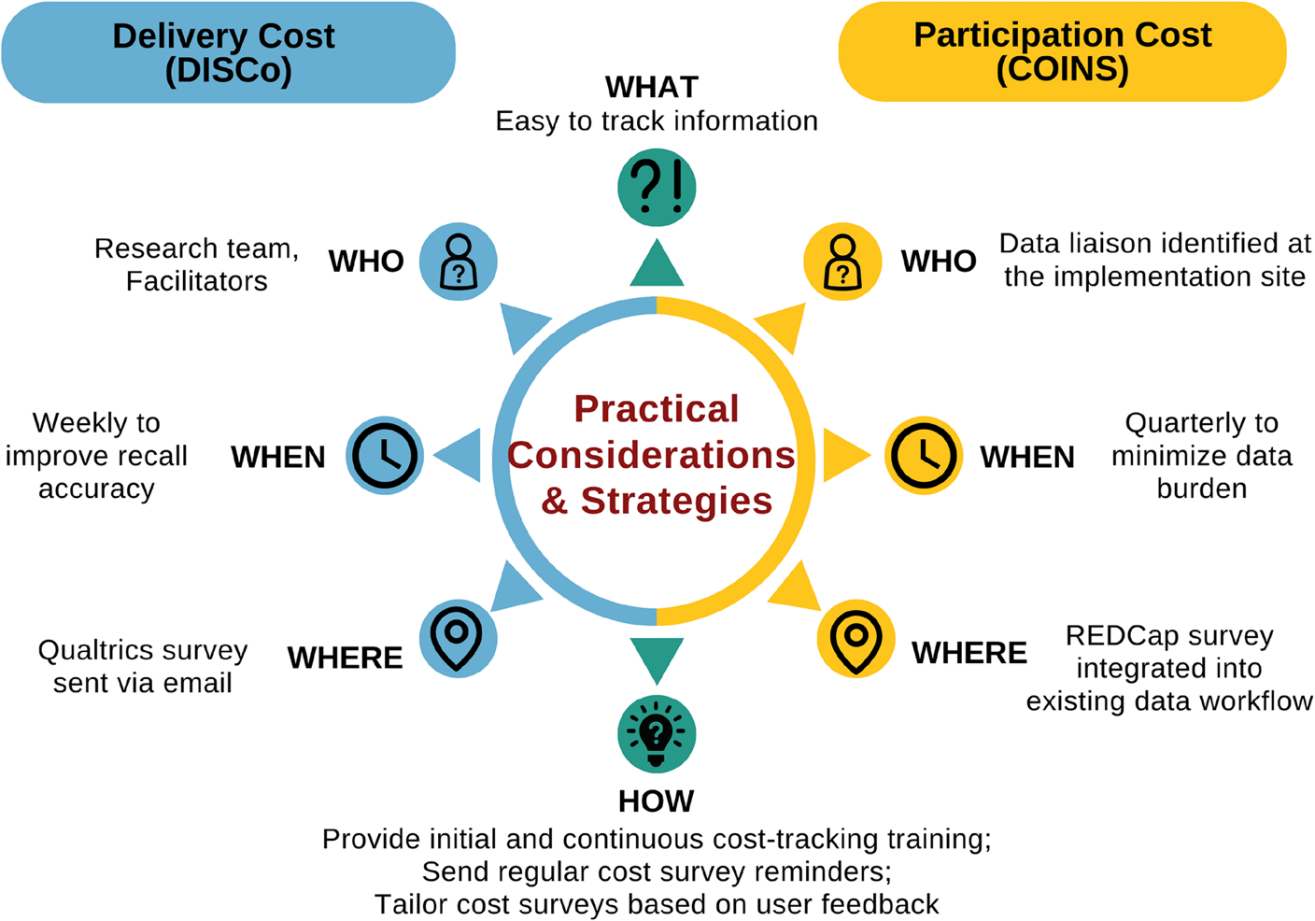
Comparison of the approach and practical considerations for mapping costs to deliver and participate in implementation strategies. Note: This figure conceptualizes implementation cost as the combination of delivery and participation costs. Delivery cost was mapped following the Delivering Implementation Strategy Cost (DISCo) guide, while participation cost was mapped utilizing the Cost of Implementing New Strategies (COINS) tool

Micro-costing entails collecting detailed data on activities and resources utilized and estimating total costs using unit costs [20, 21]. Given SITT-MAT economic evaluation’s focus on documenting implementation strategy cost, only implementation costs, as described by Gold et al. [1], were tracked and analyzed in this study. We separately measured, as noted earlier, the resources used to *deliver or participate* in the implementation strategies (Fig. 2). Intervention costs associated with MOUD service delivery, downstream costs for health care utilization (e.g., emergency room visits due to opioid overdose), and research costs to collect data for scientific evaluation were excluded. Table 1 describes cost categories and their application in SITT-MAT.

**Table 1:** Cost categories and application in SITT-MAT cost mapping

Cost Categories	Definition	Application in SITT-MAT
Implementation Costs	Resources used to execute the implementation strategy.	Resources used to deliver and participate in the sequence of four implementation strategies.
Research Costs	Costs associated with research activities that will not take place in usual practice.	[Excluded] Resources for research data collection. Note: There is a grey area here – that is when the implementation strategy is A&F. Effort to collect research data that are part of the A&F strategy is considered implementation costs.
Direct Implementation Costs	Resources directly involved in implementation.	Resources *directly* involved in delivering and participating in the sequence of four implementation strategies, categorized into o*ne time costs* and *recurring costs.*
Overhead Implementation Costs	Resources that support an organization’s implementation efforts,but are not directly involved in the services offered. Examples are human resources, information technology, administrative fees.	Resources *indirectly* involved; 30% healthcare system costs [22].
Intervention Costs	Changes in healthcare and non-healthcare resources that are the target of the implementation efforts. This can include patient and caregiver time.	[Excluded] Resources and time for MOUD.
Downstream Costs	Changes in resource utilization as a result of the implementation (e.g., healthcare utilization, productivity of patient and caregiver). These exclude the intervention costs.	[Excluded] E.g., Resources for emergency room visits as a result of opioid overdose.

Implementation costs include *direct* costs, which are the resources directly involved in *delivering* and *participating* in the sequence of four implementation strategies. These direct costs can be one-time costs or they can recur. In SITT-MAT, direct costs were presented as one-time costs and recurring costs. One-time costs refer to the upfront cost that will not be incurred again throughout the implementation period and typically do not vary with the scale of production. Recurring costs repeat and can *vary* over time (e.g., number of providers and staff present at the strategy offered). Table 2 describes the breakdown of the one-time and recurring cost elements by implementation strategy in SITT-MAT. Steps 2 and 3 outline the *how* of capturing these direct costs.

**Table 2:** Breakdown of one-time and recurring costs by implementation strategies

Implementation Strategies	Definition	Cost to Deliver Implementation Strategy		Cost to Participate in Implementation Strategy	
		One-Time Cost	Recurring Cost	One-Time Cost	Recurring Cost
Audit and Feedback (A&F)	Consists of gathering RE-AIM-guided performance data from participating clinics and presenting to them in a quarterly dashboard with normative status.	• Labor to tailor design data collection tools and develop curriculum for the data launch webinar*. • REDCap server fee (free)	• Labor to validate quarterly data, update the dashboard quarterly, provide continuous data training.	• Labor to set up internal system to track the performance data and to attend the data launch webinar.	• Labor to report quarterly data, review quarterly dashboard, meet with team to discuss progress and identify areas for growth based on feedback, receive additional data training.
Two-Day Workshop	Consists of a two-day virtual workshop that provided participating clinics with rationale, clinical practice, and program integration with MOUD (Day 1), and an overview of NIATx process improvement principles and tools (Day 2).	• Labor to develop the workshop curriculum*. • Labor to invite speakers, deliver the didactic sessions, apply for the continuing medical education (CME) accreditation. • Administrative fee for CME accreditation	• Labor to facilitate the interactive activities in small breakout rooms.	• None	• Labor to attend and register for the workshops. • Labor to utilize the NIATx tools presented in the workshop with change team.
Internal Facilitation	Features one or more internal change leaders (clinic employees) who will complete an online course to learn about the NIATx principles. The change leader(s) help other staff gain skills and efficacy to implement changes, and address barriers in attitude and beliefs using the NIATx tools presented in the course.	• Labor to build the internal facilitation online modules*. • WordPress server fee (free).	• Labor to maintain the online modules, provide on-demand support. • Labor to hold quarterly group facilitation calls.	• Labor to identify one or more clinic employee as the internal change leader.	• Labor to complete the online modules. • Labor to complete the recommended NIATx activities with change team.
External Facilitation	Features monthly check-ins with experienced NIATx External Facilitators (coaches) to plan change projects, review ongoing change efforts, discuss successes, and offer guidance on planning future change projects using PDSA cycles.	• Labor to develop the facilitation curriculum, toolkits, and relevant resources*.	• Labor to facilitate coaching in real-time and asynchronously.	• Labor to identify an internal change team.	• Labor to schedule and attend facilitation sessions, meet with team to advance the implementation effort.

Note: Activities marked with an asterisk relate to the development of data collection tool and curriculum that will not be repeated if the same strategy is applied for the same intervention and setting. Their associated costs will be removed for the final cost-outcome comparison. REDCap and WordPress servers were free of charge from the university

Implementation costs can also include overhead costs that capture resources such as human resources, information technology, and administrative fees that support an organization’s operations. These costs are indirectly involved in the services offered. Since SITT-MAT implementation took place in addiction specialty treatment programs and primary clinics, the study opted for a more conservative estimate of 30% overhead costs as recommended in prior research to avoid overinflating the implementation cost [22]. Other studies, such as one conducted within the Veteran Health Administration [23], have presented higher overhead estimates, but they are relevant when employees work in a hospital.

#### Step 2: Measure the direct cost to deliver implementation strategies

The direct cost to deliver implementation strategies consisted of the labor of *actors,* or *strategy deliverers*, such as the research staff members, facilitators, and physician experts designing and delivering the implementation support. Using DISCo as a guide, the costs to deploy each implementation strategy were tracked along the Exploration, Preparation, Implementation, and Sustainment (EPIS) [24] phases of implementation. The Preparation and Implementation phases were the primary focus for cost-tracking as they align well with the implementation strategy development and delivery process.

A weekly Qualtrics survey was developed to capture the specific time period for reporting, activities, and time allocation associated with each strategy. Qualtrics was chosen as the survey platform to enhance the overall user experience, with easy-to-use features such as the date pickers and matrix tables. The survey was designed to capture costs associated with delivering the implementation strategies and excluded costs associated with the research activities. This approach maximized recall and minimized the potential omission of critical cost components. In addition to weekly surveys, other data sources included facilitation logs and invoices for the internal and external facilitation strategies.

Non-personnel direct costs in SITT-MAT included fees associated with the Research Electronic Data Capture (REDCap) server utilized to host the A&F data collection tool and the WordPress server to host the Internal Facilitation online module, both of which were free of charge to the project through the universities.

#### Step 3: Measure the direct cost to participate in implementation strategies

SITT-MAT worked to engage the MOUD implementation team, which may include up to 10 clinical and non-clinical staff at each site. These recipients were the target of the strategies. We estimated the cost for recipients to participate and engage with the implementation strategies. This cost predominantly included the *target recipients’ time*. Quarterly REDCap surveys were employed to collect essential cost-related information from participating clinics. REDCap was chosen to host these surveys as it had already been integrated into the existing clinic workflow for gathering A&F data. The surveys capture expenses tied to engaging with ongoing support, internal meetings, and MOUD implementation activities. A data liaison was identified and trained at each clinic to complete the survey. This data collection approach was found effective in a prior implementation program in safety-net primary care clinics [25]. Informal interviews and check-ins with clinic staff were performed to validate the accuracy and assess data missing. Other data sources included attendance trackers and the internal facilitation online portal that tracked clinic staff participation across implementation strategies.

We tracked recipients’ progress along the Stages of Implementation Completion (SIC^®^) [26] utilizing its companion cost mapping tool, the Cost of Implementing New Strategies (COINS) [14]. The SIC is an 8-stage, 3-phase assessment tool that is psychometrically valid and reliable [26–30]. The SIC is designed to measure and compare implementation strategies for scaling up proven interventions [31]. Stages range from engagement (Stage 1) to achievement of program delivery with competency (Stage 8). SIC data include a log of activities that operationalize the implementation process necessary to move toward successful program start-up and sustainment and their completion dates. As a companion to the SIC, COINS is a cost mapping tool to document *implementation costs* (the cost for *target recipients* to participate in the four implementation strategies offered), which is distinct from *intervention costs* (the cost of MOUD and associated service delivery in this case) [14].

#### Step 4: Calculate the labor costs using activity data

Costs can be estimated using the personnel efforts documented in Step 2 and Step 3. We generated hours of activity data and then used national median wages from the Bureau Labor of Statistics (BLS) [32], based on the personnel’s position title and qualifications. In some cases, activities were completed by staff who were overqualified because this was a research trial. In such instances, costs were estimated for a role with the qualifications necessary to carry out the activities in routine practice. This process was done in consultation with the clinics. An additional 30% of fringe for employee benefits and 30% of fringe for overhead costs were applied, as previously described [22].

### Practical considerations

Micro-costing can be a tedious and time-consuming process that entails direct measurements from all personnel involved. In the pursuit of comprehensive and reliable cost estimation, the following considerations were applied to enhance the precision and accuracy of cost data.

#### Balance the frequency and length of cost surveys

Too frequent surveys can lead to survey fatigue and potential inaccuracies, while infrequent surveys might result in recall biases [33]. By carefully determining the optimal survey intervals and content, this cost-mapping approach captured cost data accurately without overburdening those involved.

#### Cost tracking training

At study start, comprehensive training was provided to the strategy delivery team and at least one staff member from each implementation clinic on effective cost-tracking practices. This training highlighted the significance of cost data, and how to accurately identify cost components and consistently document their efforts.

#### Regular survey reminders

Email reminders were sent to strategy deliverers (weekly) and recipients (quarterly) to encourage consistent participation in cost surveys. These reminders ensured that cost data were captured in a timely manner, minimizing potential errors.

#### Tailoring cost surveys

By incorporating user feedback, the cost surveys were tailored to align with the needs and preferences of those providing cost data. This strategy ensured the collected information was relevant, comprehensive, and reflective of the actual resource allocation.

#### Perform frequent cost data validation

To assess missingness, accuracy, and quality of the cost data collected, regular “inventory” checks were completed by the cost-mapping team (usually, this would be the analyst) approximately once a month to identify discrepancies and enable prompt corrections. Personnel and/or clinics with missed entries were prompted via email to submit the survey as soon as feasible. Meeting times across the project team were cross-checked to ensure the efforts of all personnel involved were accurately captured. This validation process was streamlined using R to maximize time savings.

#### Iterative evaluation and refinement

Insights gained from evaluating the accuracy and missingness of cost data were used to refine the data collection approach, thus improving the precision and reliability of the final cost estimates. For example, one refinement involved better defining the activities tracked in the cost survey to ensure respondents have a shared understanding of what should be included in each category.

Figure 2 summarizes the approach and practical considerations to collect costs to deliver and participate in implementation strategies.

## Results

### Case example of implementation cost of A&F

The first of a sequence of four implementation strategies in SITT-MAT was A&F, which is widely used across healthcare settings [7]. The A&F incorporated the use of performance data, which were compiled and reflected back to participating clinics with normative status [6]. It addresses contextual barriers at the system and organizational level [3]. Table 3 describes the clinic-reported RE-AIM performance data collected as part of A&F [35, 36]. Supplementary File 2 provides an example of the quarterly A&F report.

**Table 3:** RE-AIM performance data for Audit and Feedback (A&F)

RE-AIM Outcome	Definition
Reach	The proportion of program patients with OUD and receiving MOUD (buprenorphine, naltrexone) within the index month.
Effectiveness	*Access*: The proportion of patients prescribed MOUD who start the medication within 72 h of OUD diagnosis. For patients requiring detoxification for naltrexone, *Access* will be operationalized as the proportion of patients who start the medication within 72 h of when it is safe. *Retention/Engagement*: The proportion of patients who have had two or more outpatient clinical visits within 34 days of their MOUD initiation visit.
Adoption	The number of onsite integrated providers who have prescribed MOUD to at least one patient within the index month.
Implementation	Changes in the Integrating Medications for Addiction Treatment (IMAT) Index total score at the beginning and end of each implementation strategy. Rated on a scale from “1-Not Integrated”, “3-Partially Integrated”, and “5-Fully Integrated”, the IMAT is a validated measure comprised of 45 items clustered into seven dimensions [34]. It integrates MOUD guidelines, expert consensus recommendations, the OUD care cascade, and best practices into a team assessment of current MOUD capability.
Maintenance	To assess sustainment, the primary outcomes detailed above will be monitored quarterly, even as an organization moves into the sustainment phase. IMAT data collection for the organizations in the sustainment phase will follow the same timeline as that of organizations still engaged in the active implementation (post each strategy, and at 1-year follow-up).

The A&F is an ideal candidate to serve as the proof-of-concept for this microcosting approach because it is a low-intensity, hands-off strategy that necessitates deliberate tracking of participation that cannot be easily observed via surveys. Participation in A&F is also often conflated with research data collection, making it crucial to demonstrate how to disentangle the two in the tracking process. Successfully applying the microcosting approach to A&F can illustrate the feasibility of estimating implementation costs across more hands-on, clearly defined implementation strategies.

### Identify and capture delivery and participation cost of A&F

#### Delivery cost

To capture the delivery cost of A&F, specific implementation activities relevant to its design and delivery were identified and outlined at the study start in collaboration with the study team. Key set-up activities for the A&F included designing the dashboard layout, planning meetings, and generating a training curriculum for data launch. Annual recurring activities included providing continuous data training and support, updating quarterly dashboards, and maintenance meetings. The A&F delivery team documented their efforts on relevant activities via a weekly Qualtrics survey (Supplementary File 3).

#### Participation cost

Key clinic activities during the launch of the A&F strategy included attending the data launch webinar, identifying a data liaison, and setting up an internal system to track the performance data. Annual recurring activities included reporting quarterly performance data, internal meetings to review the quarterly dashboard, and engaging with additional data training and support. The clinic staff documented their efforts on relevant activities via a quarterly REDCap survey (Supplementary File 4). Examples of survey questions to capture clinic participation were: "How long (in minutes) did it take your team to complete the MOUD Program Measures in the past quarter?" and "Which of the following staff (including yourself) participated in the completion of the MOUD Program Measures in the past quarter?" These questions captured both the activity duration and personnel's role. In this case example, we used COINS to guide the estimation of participation costs, but cost estimations were not derived directly from the SIC/COINS system. This approach serves as a proof-of-concept, allowing researchers and practitioners without access to COINS to estimate costs using similar methods.

### Implementation costs of A&F

**Fig. 3:**
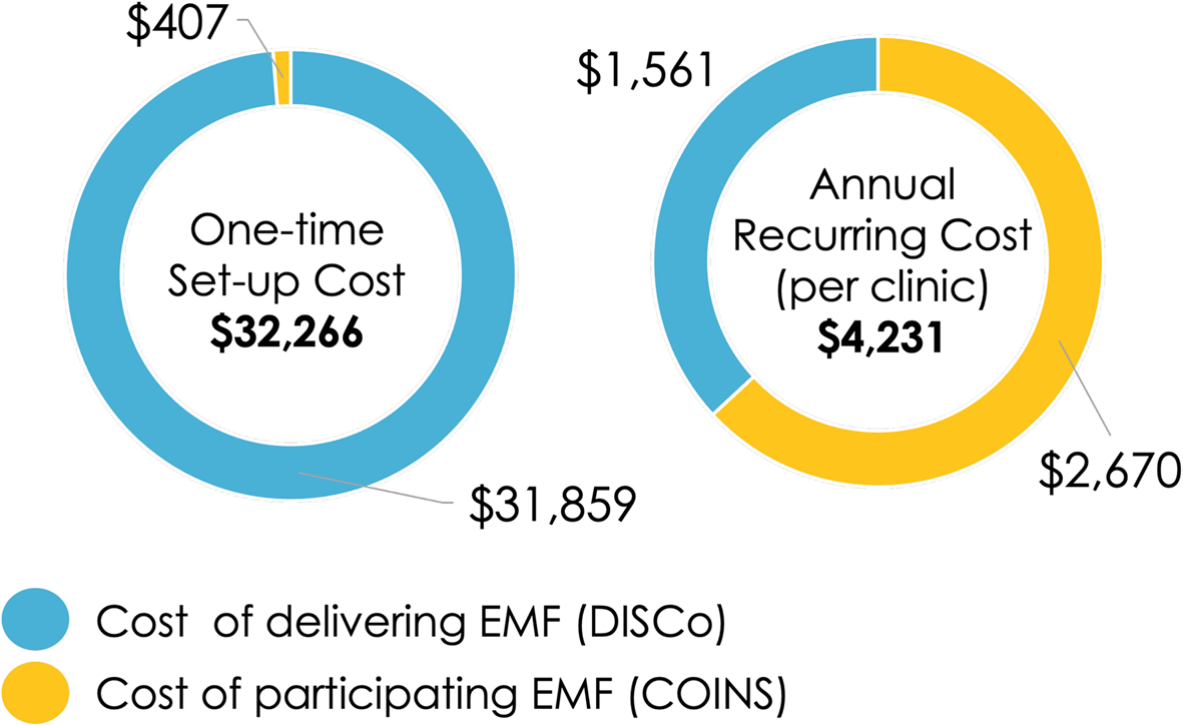
Implementation cost estimates of Audit and Feedback (A&F)

Personnel efforts were converted to cost estimates using the 2022 BLS database. Across clinics, A&F incurred an implementation setup cost of $32,266, with most of these costs attributed to the development of the A&F dashboard. The annual recurring cost per clinic was $4,231, with approximately 63% of the cost attributed to the clinic’s participation in the A&F strategy and the remaining 37% as the cost of maintaining the quarterly data dashboard. Figure 3 summarizes the one-time and annual recurring costs of the A&F strategy. Tables 4 and 5 detail the cost calculations by implementation activities**.**

**Table 4:** Delivery cost estimates of Audit and Feedback (A&F)

Cost Category	Activity	Cost
One-Time	Developing performance measures collection tool	$12,065
	Designing dashboard layout	$11,458
	Planning meetings	$5,484
	Generating training curriculum for data launch	$2,852
	Subtotal	$31,859
Recurring	Providing continuous data training & support	$856
	Updating quarterly dashboard (including data validation)	$498
	Maintenance meetings	$207
	Subtotal (per year, per site)	$1,561
	Annual total	$33,420

**Table 5:** Participation cost estimates of Audit and Feedback (A&F)

Cost Category	Activity	Cost
One-Time	Attending the data launch webinar	$271
	Identifying a data liaison	$68
	Setting up internal system to track the performance data	$68
	Subtotal	$407
Recurring	Reporting quarterly data	$1,524
	Meeting internally to review quarterly dashboard	$1,011
	Engaging with additional data training/support	$135
	Subtotal (per year, per site)	$2,670
	Annual total	$3,077

### Completeness of the cost data

Data completeness was monitored by examining response rates for the weekly delivery cost survey and quarterly participation cost surveys. The A&F delivery team demonstrated excellent response rates, with fewer than 10% of late responses and no missing data. Late responses tended to be submitted shortly after reminder nudges were sent. In contrast, adherence to the participation cost surveys was more variable. Some clinics were late adopters of A&F and thus did not complete the survey. Others did not complete the survey due to data collection burden, which was not unique to cost data collection but observed across other aspects of the research protocol. Clinics with a strong study champion or lower staff turnover had consistently higher response rates.

## Discussion

The cost-mapping approach presented here is a pragmatic and meaningful method for documenting total implementation costs. This simple approach provides important cost information so that health system and organization-level leaders can make informed, data-based decisions before embarking on a desired implementation endeavor. Considering the costs of obtaining the necessary implementation support and the requirements for staff time beforehand are critical.

We applied this cost-mapping approach to A&F to demonstrate its utility in a hands-off, low-intensity strategy that required deliberate tracking of participation. Through this application, we learned that offering A&F does not guarantee engagement. As noted in prior literature [37], assessing barriers and drivers of A&F use should be prioritized during the design and development of the strategy. The A&F reports should also be simplified and displayed in multiple formats (i.e., graphic, text, numerical), as well as delivered both in written and verbal formats [6, 37]. When delivering A&F in low-resource settings where automated data extraction from the electronic health record is not feasible, A&F can be time-intensive and place data collection burden on clinicians and staff, thus potentially undermining its effectiveness. When estimating the cost of A&F, mechanisms should be put in place to distinguish between efforts to collect data for research evaluation and efforts to collect data for A&F to maximize accuracy of the cost estimation.

The same cost-mapping approach can also be generalized for higher-intensity, more hands-on strategies. Rather than relying on strategy recipients to document their participation via surveys, the strategy deliverers, such as facilitators or workshop hosts, can track attendance directly. Regardless of whether the strategy is low or high intensity, the key is to proactively identify the individual(s) who will document participation and determine how this documentation will take place (e.g., via surveys, facilitation logs, etc.). Moreover, when working with multiple strategies simultaneously, efforts should be made to clearly define the components of each ahead of implementation to avoid overlaps or conflation.

While this micro-costing approach shares similarities to the time-driven activity-based costing that details costs associated with implementation strategies based on actions, temporality, and the actors involved [15], strengths of this approach include the checks and balances used to gather cost data with high accuracy and sufficient precision. The conceptualization of implementation cost as the combination of *delivery* and *participation* cost offers well-defined perspectives on the incurred costs. The intentional call out of implementation strategy delivery cost using the DISCo complements the SIC [38] and its companion tool COINS, which primarily focuses on capturing implementation participation cost from the perspective of implementation sites [10]. Similar to the SIC and COINS, DISCo measures delivery costs, and when measured precisely over time, it can be used to measure costs that align with the four EPIS phases [24], from pre-implementation (exploration, preparation) to implementation and sustainment [4]. Despite traditionally operating as separate fields, health economics and implementation science are increasingly intersecting. This paper begins to bridge the two fields by fostering a shared understanding of cost categories in implementation efforts, which aligns with the recommendations from a recent scoping review [12].

Although this pragmatic micro-costing approach is overall useful and relatively simple to follow, there are a few limitations. Due to their competing priorities, some implementation sites did not consistently record their implementation activities and engagement via surveys. Because clinics with strong study champions demonstrated better response rates, efforts should be made early on to build rapport with clinics, co-design data collection forms with their inputs, train the study champion with data collection, and identify backup in case of staff turnover. Future cost data collection efforts should also focus on sending requests and reminders during non-holiday weeks to minimize low adherence. Cost-relevant notes from progress meetings with coaches and qualitative interviews can be utilized to supplement the quantitative survey data [39]. Moreover, the accuracy and precision of cost data may be higher for more “hands-on” implementation strategies, such as internal and external facilitation. Given this manuscript primarily focuses on how to apply a micro-costing approach to estimate implementation costs, rather than interpretation of the cost findings, sensitivity analyses were not performed. Sensitivity analyses can be applied during cost-outcome analyses to examine uncertainties [40]. Finally, the SITT-MAT study did not consider intervention and downstream costs as those outcomes were beyond the scope this proposed work. Such costs should be investigated in future studies to better understand the long-term cost-benefits of implementation strategies and efforts.

For the next step in advancing this micro-costing approach, our team will utilize the cost data collected to present the implementation delivery and participation cost of each strategy. We will also examine cost and outcomes by contextual factors (e.g., primary care vs. specialty care, rural vs. urban/metropolitan, size of implementation team) to understand contextual differences. The breakdown of implementation outcome and cost by strategy and contextual factors can aid implementation decision-makers in identifying the most appropriate strategy given a public health goal, budget, and setting. Efficiency analysis will then be conducted using an output maximization approach with data envelopment analysis [41] or cost minimization approach using stochastic frontier analysis [41]. The findings will help identify efficient models that can be adopted by implementation sites with similar characteristics to ensure the limited resources can have the widest reach.

There is promising value in testing the generalizability of this pragmatic micro-costing approach in other settings. A recent qualitative study highlighted the need to standardize the measurement of implementation cost to improve transparency and confidence in implementation cost estimates [13]. The pragmatic micro-costing approach presented here has the potential to address this measurement challenge if proven useful across settings. As more studies capture implementation costs and outcomes, the field can accumulate a database to improve cost transparency and efficiency in implementation planning.

## Conclusion

This implementation cost-mapping approach and the companion practical considerations could help implementation decision-makers, researchers, and practitioners in selecting costing procedures that align with their needs. Future research should test the utility and generalizability of this approach with other health interventions and healthcare settings. By understanding the total cost implications of implementation, decision-makers could better select the most suitable strategy based on the context, goals, timeline, and budget constraints.

## Supplementary Information

Supplementary Material 1.

Supplementary Material 2.

Supplementary Material 3.

Supplementary Material 4.

## Data Availability

The datasets generated during and/or analyzed during the current study are not publicly available because data belong to the participating clinics but are available from the corresponding author on reasonable request.

## Abbreviations

A&F: Audit and Feedback
CHEERS: Consolidated Health Economic Evaluation Reporting Standards
COINS: Cost of Implementing New Strategies
DISCo: Delivering Implementation Strategy Cost
EPIS: Exploration, Preparation, Implementation, and Sustainment
MOUD: Medications for Opioid Use Disorder
OUD: Opioid Use Disorder
RE-AIM: Reach, Effectiveness, Adoption, Implementation, Maintenance
REDCap: Research Electronic Data Capture
SIC: Stages of Implementation Completion
SITT-MAT: Stagewise Implementation-To-Target – Medications for Addiction Treatment

## References

[1] Gold HT, McDermott C, Hoomans T, Wagner TH. Cost data in implementation science: categories and approaches to costing. Implement Sci. 2022;17(1):11. 35090508 10.1186/s13012-021-01172-6 PMC8796347

[2] Proctor EK, Powell BJ, McMillen JC. Implementation strategies: recommendations for specifying and reporting. Implement Sci. 2013;8(1):139. 24289295 10.1186/1748-5908-8-139 PMC3882890

[3] Ford JH, Cheng H, Gassman M, Fontaine H, Garneau HC, Keith R, et al. Stepped implementation-to-target: a study protocol of an adaptive trial to expand access to addiction medications. Implement Sci. 2022;17(1):64. 36175963 10.1186/s13012-022-01239-y PMC9524103

[4] Saldana L, Ritzwoller DP, Campbell M, Block EP. Using economic evaluations in implementation science to increase transparency in costs and outcomes for organizational decision-makers. Implement Sci Commun. 2022;3(1):40. 35410434 10.1186/s43058-022-00295-1 PMC9004101

[5] Hoomans T, Severens JL. Economic evaluation of implementation strategies in health care. Implement Sci. 2014;9(1):1–6. 25518730 10.1186/s13012-014-0168-y PMC4279808

[6] Ivers N, Jamtvedt G, Flottorp S, Young JM, Odgaard-Jensen J, French SD, et al. Audit and feedback: effects on professional practice and healthcare outcomes. Cochrane Database Syst Rev. 2012. Available from: http://doi.wiley.com/10.1002/14651858.CD000259.pub3. 10.1002/14651858.CD000259.pub3 PMC11338587 22696318

[7] Ashcraft LE, Goodrich DE, Hero J, Phares A, Bachrach RL, Quinn DA, et al. A systematic review of experimentally tested implementation strategies across health and human service settings: evidence from 2010–2022. Implement Sci. 2024;19(1):43. 38915102 10.1186/s13012-024-01369-5 PMC11194895

[8] Eisman AB, Quanbeck A, Bounthavong M, Panattoni L, Glasgow RE. Implementation science issues in understanding, collecting, and using cost estimates: a multi-stakeholder perspective. Implement Sci. 2021;16(1):1–12. 34344411 10.1186/s13012-021-01143-x PMC8330022

[9] Waller G, Ferguson J, Bray JW, Newbury-Birch D, Stoddart A, Holloway A. Implementation Costs of the APPRAISE Alcohol Brief Intervention (ABI) for Male Remand Prisoners: A Micro-Costing Protocol and Preliminary Findings. J Stud Alcohol Drugs. 2024;85(6):820–8. 38669136 10.15288/jsad.23-00341

[10] Bowser DM, Henry BF, McCollister KE. Cost analysis in implementation studies of evidence-based practices for mental health and substance use disorders: a systematic review. Implement Sci. 2021;16(1):26. 33706780 10.1186/s13012-021-01094-3 PMC7953634

[11] Reeves P, Edmunds K, Searles A, Wiggers J. Economic evaluations of public health implementation-interventions: a systematic review and guideline for practice. Public Health. 2019;169:101–13. 30877961 10.1016/j.puhe.2019.01.012

[12] Malhotra A, Thompson RR, Kagoya F, Masiye F, Mbewe P, Mosepele M, et al. Economic evaluation of implementation science outcomes in low- and middle-income countries: a scoping review. Implement Sci. 2022;17(1):76. 36384807 10.1186/s13012-022-01248-x PMC9670396

[13] Donovan T, Carter HE, McPhail SM, Abell B. Challenges and recommendations for collecting and quantifying implementation costs in practice: a qualitative interview study. Implement Sci Commun. 2024;5(1):1–15. 39394175 10.1186/s43058-024-00648-y PMC11468373

[14] Saldana L, Chamberlain P, Bradford WD, Campbell M, Landsverk J. The Cost of Implementing New Strategies (COINS): A Method for Mapping Implementation Resources Using the Stages of Implementation Completion. Child Youth Serv Rev. 2014;1(39):177–82. 10.1016/j.childyouth.2013.10.006 PMC3979632 24729650

[15] Cidav Z, Mandell D, Pyne J, Beidas R, Curran G, Marcus S. A pragmatic method for costing implementation strategies using time-driven activity-based costing. Implement Sci. 2020;15(1):1–15. 32370752 10.1186/s13012-020-00993-1 PMC7201568

[16] Sohn H, Tucker A, Ferguson O, Gomes I, Dowdy D. Costing the implementation of public health interventions in resource-limited settings: a conceptual framework. Implement Sci. 2020;15(1):1–8. 32993713 10.1186/s13012-020-01047-2 PMC7526415

[17] Morris ZS, Wooding S, Grant J. The answer is 17 years, what is the question: understanding time lags in translational research. J R Soc Med. 2011;104(12):510–20. 22179294 10.1258/jrsm.2011.110180 PMC3241518

[18] Proctor EK, Powell BJ, McMillen JC. Implementation strategies: recommendations for specifying and reporting. Implement Sci. 2013;1(8):139. 10.1186/1748-5908-8-139 PMC3882890 24289295

[19] Husereau D, Drummond M, Petrou S, Carswell C, Moher D, Greenberg D, et al. Consolidated Health Economic Evaluation Reporting Standards (CHEERS) statement. Cost Eff Resour Alloc. 2013;11(1):1–6. 23531194 10.1186/1478-7547-11-6 PMC3607888

[20] Gold MR. Cost-Effectiveness in Health and Medicine. USA: Oxford University Press; 1996. p. 462.

[21] Frick KD. Microcosting quantity data collection methods. Med Care. 2009;47(7 Suppl 1):S76-81. 19536026 10.1097/MLR.0b013e31819bc064 PMC2714580

[22] Anderson RJ. Reducing and Controlling Overhead Costs. Drug Inf J. 1996;30(1):89–96.

[23] Determination of VA Health Care Costs - Paul G. Barnett, 2003. Available from: https://journals-sagepub-com.stanford.idm.oclc.org/doi/10.1177/1077558703256483?icid=int.sj-abstract.similar-articles.1. Cited 9 Feb 2025. 10.1177/1077558703256483 15095549

[24] Aarons GA, Hurlburt M, Horwitz SM. Advancing a conceptual model of evidence-based practice implementation in public service sectors. Adm Policy Ment Health. 2011;38(1):4–23. 21197565 10.1007/s10488-010-0327-7 PMC3025110

[25] Cheng H, McGovern MP, Garneau HC, Hurley B, Fisher T, Copeland M, et al. Expanding access to medications for opioid use disorder in primary care clinics: an evaluation of common implementation strategies and outcomes. Implementation Science Communications. 2022;3(1):72. 35794653 10.1186/s43058-022-00306-1 PMC9258188

[26] Saldana L. The stages of implementation completion for evidence-based practice: protocol for a mixed methods study. Implement Sci. 2014;9(1):43. 24708893 10.1186/1748-5908-9-43 PMC4234147

[27] Saldana L, Chamberlain P. Supporting implementation: the role of community development teams to build infrastructure. Am J Community Psychol. 2012;50(3–4):334–46. 22430709 10.1007/s10464-012-9503-0 PMC3397152

[28] Chamberlain P, Brown CH, Saldana L. Observational measure of implementation progress in community based settings: the Stages of Implementation Completion (SIC). Implement Sci. 2011;6(6):116. 21974914 10.1186/1748-5908-6-116 PMC3197550

[29] Chamberlain P, Roberts R, Jones H, Marsenich L, Sosna T, Price JM. Three Collaborative Models for Scaling Up Evidence-Based Practices. Adm Policy Ment Health. 2012;39(4):278–90. 21484449 10.1007/s10488-011-0349-9 PMC4312010

[30] Saldana L, Schaper H, Campbell M, Chapman J. Standardized measurement of implementation: the universal SIC. Implement Sci. 2015;10(1):1–1.25567289

[31] Brown CH, Chamberlain P, Saldana L, Padgett C, Wang W, Cruden G. Evaluation of two implementation strategies in 51 child county public service systems in two states: results of a cluster randomized head-to-head implementation trial. Implement Sci. 2014;9(1):1–15. 25312005 10.1186/s13012-014-0134-8 PMC4201704

[32] OES Home : U.S. Bureau of Labor Statistics. Available from: https://www.bls.gov/oes/. Cited 15 Nov 2023.

[33] Neumann PJ, Ganiats TG, Russell LB, Sanders GD, Siegel JE, editors. Cost-Effectiveness in Health and Medicine. Oxford University Press; 2016. Available from: 10.1093/acprof:oso/9780190492939.001.0001. Cited 28 Jul 2025.

[34] Chokron Garneau H, Hurley B, Fisher T, Newman S, Copeland M, Caton L, et al. The integrating medications for addiction treatment (IMAT) index: a measure of capability at the organizational level. J Subst Abuse Treat. 2021;126:108395. 34116810 10.1016/j.jsat.2021.108395

[35] Glasgow RE, Harden SM, Gaglio B, Rabin B, Smith ML, Porter GC, et al. RE-AIM Planning and Evaluation Framework: Adapting to New Science and Practice With a 20-Year Review. Front Public Health. 2019;7. Available from: https://www.frontiersin.org/articles/10.3389/fpubh.2019.00064/full. Cited 3 Jan 2021. 10.3389/fpubh.2019.00064 PMC6450067 30984733

[36] Glasgow RE, Vogt TM, Boles SM. Evaluating the public health impact of health promotion interventions: the RE-AIM framework. Am J Public Health. 1999;89(9):1322–7. 10474547 10.2105/ajph.89.9.1322 PMC1508772

[37] Brehaut JC, Colquhoun HL, Eva KW, Carroll K, Sales A, Michie S, et al. Practice Feedback Interventions: 15 Suggestions for Optimizing Effectiveness. Ann Intern Med. 2016;164(6):435–41. 26903136 10.7326/M15-2248

[38] Dopp AR, Mundey P, Beasley LO, Silovsky JF, Eisenberg D. Mixed-method approaches to strengthen economic evaluations in implementation research. Implement Sci. 2019;14(1):2. 30635001 10.1186/s13012-018-0850-6 PMC6329154

[39] Sensitivity Analysis in Decision Making on JSTOR. Available from: https://www.jstor.org/stable/243710?casa_token=j7UHQK9PNvsAAAAA%3A3yJp1avj2-1yRidECEwJO2hgluM8M1noELT6xgl5MQT2vGdK8VgXTJWSkrj1mhfZr4PIxzxziDNULyHZ68kMeMF3ER3Rqw2P2JNjFdkbn3KSOwekAMfg3w&seq=1. Cited 11 Feb 2025.

[40] Pluta K, Hohl SD, D’Angelo H, Ostroff JS, Shelley D, Asvat Y, et al. Data envelopment analysis to evaluate the efficiency of tobacco treatment programs in the NCI Moonshot Cancer Center Cessation Initiative. Implement Sci Commun. 2023;4(1):50. 37170381 10.1186/s43058-023-00433-3 PMC10173908

[41] Bala MM, Singh S, Gautam DK. Stochastic frontier approach to efficiency analysis of health facilities in providing services for non-communicable diseases in eight LMICs. Int Health. 2023;15(5):512–25. 36515155 10.1093/inthealth/ihac080 PMC10472875

