# Supplementary material for: A pragmatic approach to estimating the cost to deliver and participate in implementation strategies
Source: Implement Sci. 2025 Oct 17;20:44. doi: 10.1186/s13012-025-01459-y (PMC12535059; doi:10.1186/s13012-025-01459-y)
Supplement: Supplementary file 2 — Supplementary Material 2. [file 13012_2025_1459_MOESM2_ESM.pdf]

Sesame Street Clinic

Quarterly Dashboard: February 29th, 2024

INTEGRATING MEDICATIONS FOR ADDICTION TREATMENT (IMAT) INDEX

The **IMAT Total** increased from 2.49 to 3.57. Biggest gain was observed in **Patient Identification & Initiating Care**.

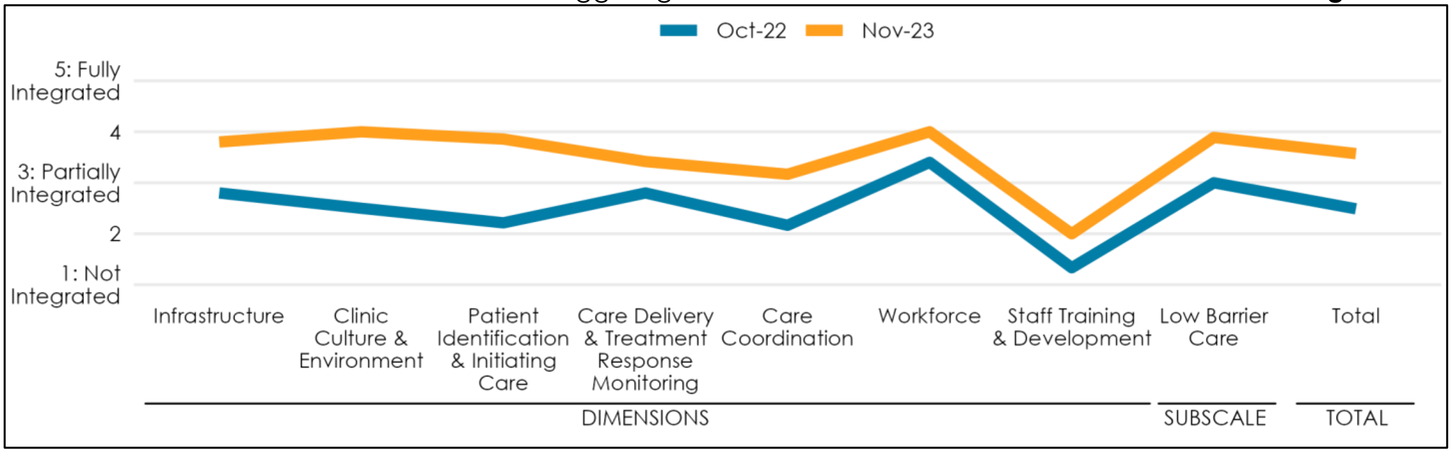

PROGRAM MEASURES

Percent of **new patients prescribed MOUD** increased from 0% (0 of 0 patients) to 50% (2 of 4 patients).

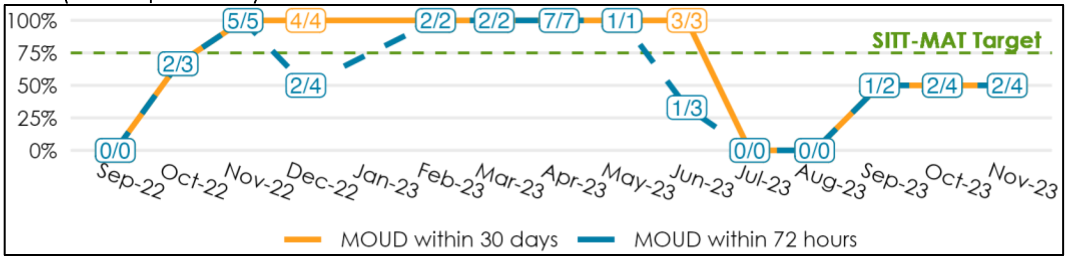

Percent of **new patients prescribed MOUD who had 2+ clinical visits within 34 days** decreased from 100% (3 of 3 patients) to 50% (2 of 4 patients).

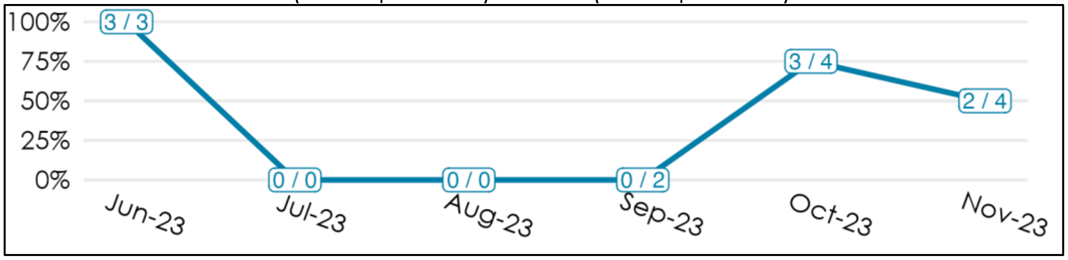

Percent of **OUD patients referred & linked** increased from 0% to 100%.

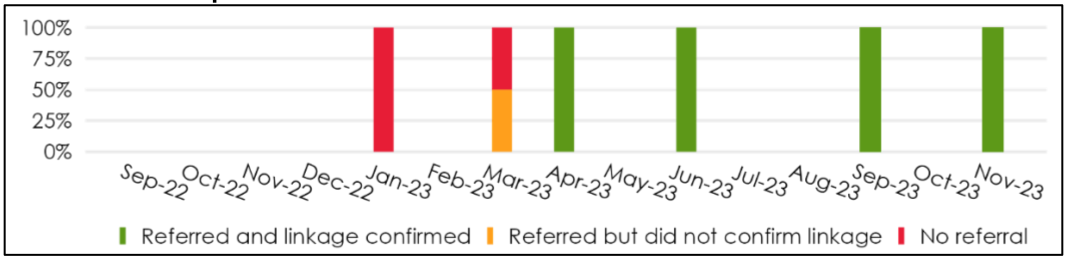

WHAT CAN YOU DO NEXT?

STEP 1:
Review the dashboard with your team.

STEP 2:
Identify any of the 7 IMAT dimensions you might seek to improve, then **select** items from those dimensions to focus on. (See next page).

STEP 3:
Determine if the Program Measures match your clinical experience, then **identify** if any changes are needed to your clinical process.

STEP 4:
Create an action plan with your team!

IMAT: November 2023

IMAT DIMENSION & TOTAL SCORES	
Infrastructure	3.80
Clinic Culture & Environment	4.00
Patient Identification & Initiating Care	3.86
Care Delivery & Treatment Response Monitoring	3.70
Care Coordination	3.17
Workforce	4.00
Staff Training & Development	2.00
Low Barrier Care	3.89
TOTAL	3.57

D1	INFRASTRUCTURE	Score
D1.1	Senior organizational and clinic leadership, including CEO, CMO, board and medical directors, strongly support providers prescribing MOUD in this clinic site	5
D1.2	Medical records and releases of information are privacy compliant with 42CFR and HIPAA regulations	4
D1.3	Insurers cover medical consultations and visits for management of MOUD or medical services are covered by bundled contractual rates	3
D1.4	Insurers cover MOUD (buprenorphine and naltrexone IM) or MOUD are covered by bundled contractual rates	4
D1.5	Insurers cover behavioral health services	3

D2	CLINIC CULTURE AND ENVIORNMENT	Score
D2.1	All clinic staff accept and welcome equally persons with OUD—no evidence for stigma or discrimination	3
D2.2	Open display and distribution of patient informational materials about OUD and MOUD in common areas, therapy rooms and offices	5
D2.3	Patients and services are visibly integrated in general clinic spaces and in routine operations	5
D2.4	All clinic staff believe offering MOUD to patients in this clinic is appropriate	3

D3	PATIENT IDENTIFICATION AND INITIATING CARE	Score
D3.1	All new and existing patients are screened using a standardized universal measure for opioid use risk	3
D3.2	All patients who screen positive receive a standardized indicated assessment and, if positive, an OUD diagnosis is made and documented	4
D3.3	All patients seen in this clinic on dosages of >90 mg of morphine equivalents (MMEs) for >3 months to manage chronic non-cancer pain are reviewed and evaluated for potential OUD diagnosis and appropriateness for buprenorphine	1
D3.4	A protocol for identification, diagnosis and treatment initiation exists for conditions commonly comorbid with OUD including other substance use disorders	5
D3.5	A protocol for identification, diagnosis and treatment initiation exists for conditions commonly comorbid with OUD including other psychiatric disorders such as depression, anxiety, PTSD or other mental health problems	3
D3.6	A protocol for identification, diagnosis and treatment initiation exists for infectious disease commonly comorbid with OUD, including HIV and HCV	3
D3.7	For patients diagnosed with OUD, the prescription drug monitoring clinic (PDMP) is queried	3
D3.8	For patients diagnosed with OUD, a point-of-care toxicology test is performed, i.e. urine drug screen, with built-in and/or rapid on-clinic immunoassay testing	5
D3.9	Patients with OUD are presented with clear treatment options, patient preferences are discussed, and a shared decision-making approach used	4
D3.10	Criteria for offering MOUD in the clinic are clear, they are documented in policy, patient information sheets/brochures and consent forms, and they are highly inclusive	4
D3.11	Three components are performed for all patients using MOUD: Withdrawal symptoms are evaluated, side effects are discussed, and comfort medications to treat opioid withdrawal are made available	4
D3.12	Patients choosing MOUD can be started within 72 hours (buprenorphine) or nearly after 72 hours (ER naltrexone injection)	5
D3.13	The clinic has a patient treatment agreement document that describes expectations of the clinic and of the patient on MOUD	5
D3.14	Using a protocol clear to both staff and patients, eligible patients can start the medication either in-home or in-office	5

D4	CARE DELIVERY AND TREATMENT REPONSE MONITORING	Score
D4.1	Patients started on MOUD have at least 1 follow-up visit within 14 days (2 weeks)	5
D4.2	Patients started on MOUD have at least 2 follow-up visits within 30 days (1 month)	4
D4.3	Ongoing toxicology testing, i.e. urine drug screen, is performed at least monthly, at random, and observed	3
D4.4	The prescription drug monitoring clinic (PDMP) is queried at least bi-monthly	3
D4.5	A protocol exists for random pill or film counts for patients prescribed buprenorphine	1
D4.6	A protocol exists, based on treatment response—including toxicology results and patient report of functioning—to adjust dose, frequency of visits and toxicological monitoring	3
D4.7	A systematic approach (e.g., ASAM criteria) is used to assess patient functioning and social determinants; This approach supports treatment planning which may include additional physical or behavioral health services either within this clinic clinic or offered in another setting	5
D4.8	A systematic approach, such as the ASAM criteria or Treatment Needs Questionnaire, is used to determine need for a more intensive level of care (residential, hospital) or setting (methadone clinic)	5
D4.9	Patients are neither encouraged nor required to taper or discontinue the medication after a certain period of time or once stabilized or with improved functioning	5
D4.10	Six-month retention rates of patient panel on MOUD are tracked to examine this clinic's processes	3

D5	CARE COORDINATION	Score
D5.1	The clinic uses a team-based care approach to manage patients treated with MOUD; team members may include physicians, nurse practitioners, physician assistants, nurses, behavioral health clinicians or counselors, peer specialists, or pharmacists; and with clearly defined, written roles and responsibilities for each member of the team	4
D5.2	A registry of patients on MOUD is used to track patient attendance, visit planning and treatment response	3
D5.3	With the most common health care and social service partners, the clinic has memoranda of understanding, agreements or clear understanding of methods to coordinate care, accept referrals, refer or link patients with primary care and/or specialists (e.g. addiction, psychiatry, OB/GYN) or services (e.g. DCFS, probation and parole)	3
D5.4	The clinic has a 42CFR and HIPAA compliant set of forms to exchange or release clinical information with patient consent	3
D5.5	An outreach procedure exists for patients who have not made appointments or about whom there is clinical concern (phone or home visit)	3
D5.6	Clinic leadership engages in regular meetings with other organizations in the geographic region (patient centered medical neighborhood) to troubleshoot, improve communication and strengthen the network of care	3

D6	WORKFORCE	Score
D6.1	X-waivered prescriber(s) onsite to prescribe MOUD	4
D6.2	Nursing or pharmacist personnel are onsite to manage MOUD and nursing related needs of patients; a nurse or pharmacist care manager model is used to perform activities during patient visits either in individual or group formats; there is coordination of care with other health care providers; patient and family education is provided	4
D6.3	Licensed behavioral health clinician(s) with credentials in both mental health AND addiction assessment and treatment are onsite; have expertise to conduct evaluations, individual, group and family/couples therapies; there is expertise in integrated behavioral health and in team-based primary care; either individual behavioral health clinicians have expertise in both mental health and addiction OR two or more clinicians have combined expertise	3
D6.4	Staff or volunteer affiliation with peer recovery support group network (e.g. NA, AA, MA, Al-Anon) to educate and connect patients on MOUD and their support persons to these resources	5
D6.5	Administrative support to manage registry, coordination of care, liaison with other agencies, and funders	4

D7	STAFF TRAINING AND DEVELOPMENT	Score
D7.1	X-waivered providers/prescribers and other clinicians are actively involved in CME or equivalent continuing education and other advanced learning opportunities focused on MOUD, addiction and integrated behavioral health care	2
D7.2	All non-clinical staff, including administrative and support personnel, have basic training in MOUD AND substance use disorders and treatment their treatment	2
D7.3	All staff (clinical and non-clinical) have completed training in empathy and stigma reduction for persons with substance use disorders	2

INVENTORY OF BARRIERS & FACILITATORS TO IMPLEMENTATION

The figure below illustrates the **barriers**, *things that get in the way*, and **facilitators**, *things that help*, with the implementation of MOUD as identified by your team.

The color of the circles (light grey – not important, medium grey – somewhat important, dark grey – very important) reflects the importance your team attributed to each factor.

As an example, "System level leadership support" is identified as a very important facilitator that you can continue to leverage and build on to implement MOUD.

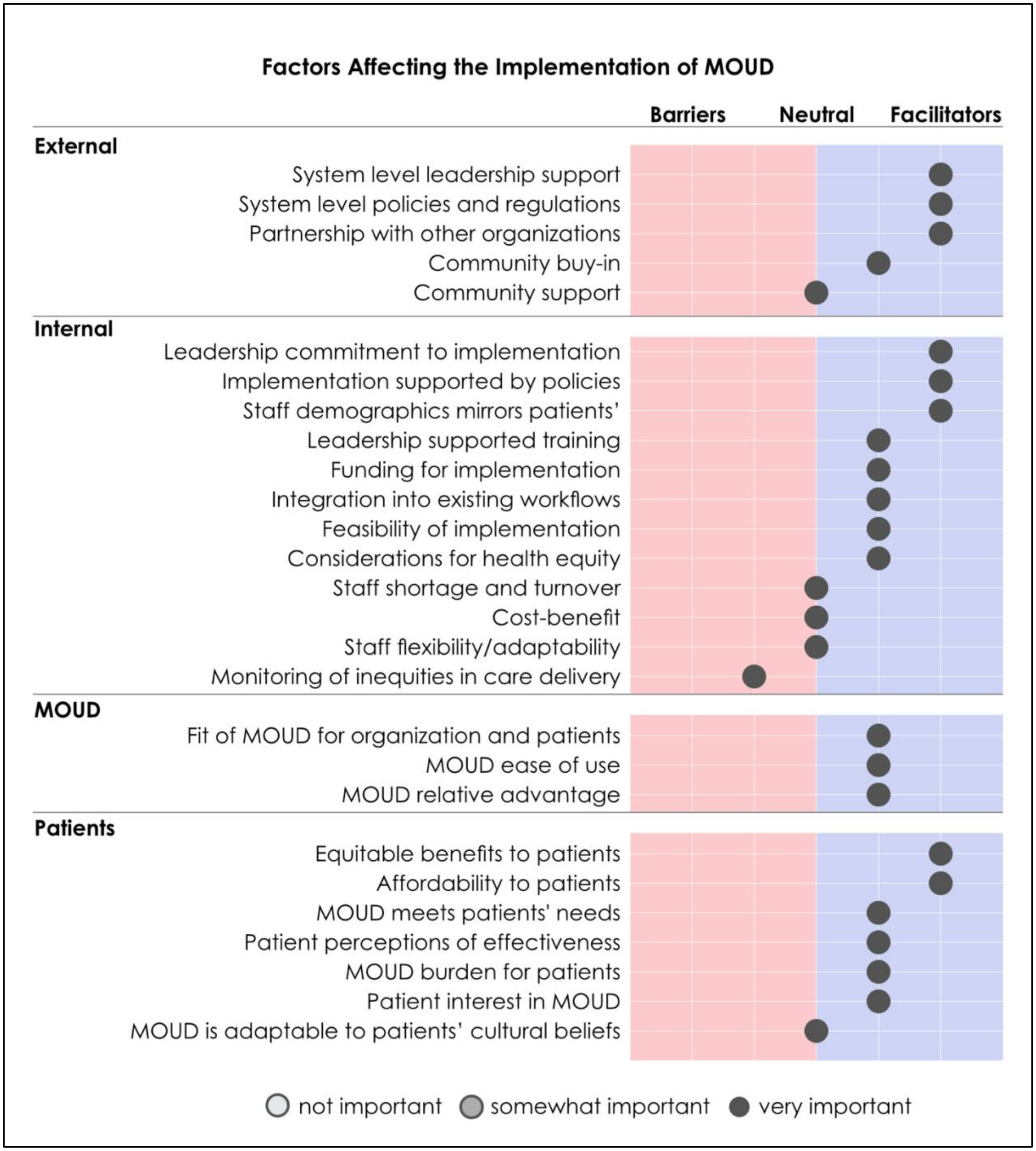

Note: Items are ordered by importance, and then scored within each dimension
