# Supplementary material for: A pragmatic approach to estimating the cost to deliver and participate in implementation strategies
Source: Implement Sci. 2025 Oct 17;20:44. doi: 10.1186/s13012-025-01459-y (PMC12535059; doi:10.1186/s13012-025-01459-y)
Supplement: Supplementary file 3 — Supplementary Material 3. [file 13012_2025_1459_MOESM3_ESM.pdf]

SITT-MAT
Stagewise Implementation To Target - Medications for Addiction Treatment

Default Question Block

INSTRUCTIONS: This survey is designed to collect data related to your time spent on SITT-MAT implementation activities for costing purposes. Please complete the following to the best of your ability. You will be prompted to complete this survey once a week.

What is your name?

[ ]

Select the START date captured by this survey:

Enter a date:

← April 2025 →

Su	Mo	Tu	We	Th	Fr	Sa
30	31	1	2	3	4	5
6	7	8	9	10	11	12
13	14	15	16	17	18	19
20	21	22	23	24	25	26
27	28	29	30	1	2	3
4	5	6	7	8	9	10

[ ]

Select the END date captured by this survey:

Enter a date:

←			April 2025			→
Su	Mo	Tu	We	Th	Fr	Sa
30	31	1	2	3	4	5
6	7	8	9	10	11	12
13	14	15	16	17	18	19
20	21	22	23	24	25	26
27	28	29	30	1	2	3
4	5	6	7	8	9	10

Overall

During the specified time period, were you involved in any SITT-MAT activities?

- Yes
- No (Note: selecting this will take you to the end of survey)

Activities

PLEASE READ: Estimate the amount of time you spent on the following activities. Leave any activities you were not involved in blank. If applicable, capture consultant efforts under optional notes.

Audit and Feedback (A&F)

	Estimated time (min)	Optional notes
Attended internal meeting(s) to discuss A&F/data collection (EXCLUDE weekly meeting on Tue)		
Designed/Reivewed/Piloted data collection instruments (i.e., IMAT, IFASIS, and MOUD Program Measures)		
Set up data submission dashboard via REDCap		
Set up A&F dashboard		
Designed/Reviewed A&F report template		

	Estimated time (min)	Optional notes
Automated calculations/visualizations of IMAT, IFASIS, and MOUD Program Measures		
Prepared for data webinar/data office hours		
Invited participants to data webinar/data office hours		
Hosted data webinar/data office hours		
Answered data relevant questions via email/phone/Zoom (outside of webinar/office hours)		
Requested IMAT, IFASIS, and MOUD Program Measures data from participants		
Validated IMAT, IFASIS, and MOUD Program Measures data (including emailed participants to gather incorrect/missing data)		
Pulled/Compiled/Analyzed IMAT, IFASIS, and MOUD Program Measures data		
Generated A&F reports (include visualizing data and drafting summary text)		
Returned A&F reports to participants		

Please describe any additional activities not listed above, specify the implementation strategy and activity, and estimate the time spent.

Additional Activity 1	
Additional Activity 2	
Additional Activity 3	
Additional Activity 4	
Additional Activity 5	

Please share any additional information that would be helpful for costing (e.g., if you were out of office for part of the week).

Powered by Qualtrics
