# Supplementary material for: A pragmatic approach to estimating the cost to deliver and participate in implementation strategies
Source: Implement Sci. 2025 Oct 17;20:44. doi: 10.1186/s13012-025-01459-y (PMC12535059; doi:10.1186/s13012-025-01459-y)
Supplement: Supplementary file 4 — Supplementary Material 4. [file 13012_2025_1459_MOESM4_ESM.pdf]

Stagewise Implementation to Target – Medications for Addiction Treatment

SITT-MAT

Survey Queue

A A A

Quarterly Implementation Activity Tracker

Instructions

Please complete the following to the best of your ability. MOUD refers to medications for opioid use disorder. Past quarter refers to 09/2022 and 11/2022

If you prefer to complete this form offline, you can download this fillable and printable PDF, complete the form, and return to this data submission portal to enter your response.

Attachment: SITT-MAT-Implementation Activity Tracker-V1.0-SC-fillable.pdf (252.8 kB)

Data Reporting for MOUD Program Measures

Did your program set up a new system OR update an existing system to collect data for the SITT-MAT MOUD Program Measures? *

- (●) Yes
- ( ) No

reset

How long (in minutes) did it take your program to set up OR update the system to collect data for the MOUD Program Measures? *

Which of the following staff (including yourself) was involved in setting up OR updating the system to collect data for the MOUD Program Measures? Please select all that apply and specify the count of each. *

- ☐ Peer Recovery Specialist, count:
- ☐ Substance Use Counselor, count:
- ☐ Mental Health Clinician, count:
- ☐ Nurse-RN/LPN, count:
- ☐ Clinical Supervisor, count:
- ☐ Prescriber-NP/PA, count:
- ☐ Prescriber-MD/DO, count:
- ☐ Program Director, count:
- ☐ Executive Director or CEO, count:
- ☐ Clinic Administrator or CCO, count:
- ☐ Medical Director or CMO, count:
- ☐ Other staff not listed above, specify role & count:

How long (in minutes) did it take your team to complete the MOUD Program Measures in the past quarter? *

Which of the following staff (including yourself) participated in the completion of the MOUD Program Measures in the past quarter? Please select all that apply and specify the count of each. *

- ☐ Peer Recovery Specialist, count:
- ☐ Substance Use Counselor, count:
- ☐ Mental Health Clinician, count:
- ☐ Nurse-RN/LPN, count:
- ☐ Clinical Supervisor, count:
- ☐ Prescriber-NP/PA, count:
- ☐ Prescriber-MD/DO, count:
- ☐ Program Director, count:
- ☐ Executive Director or CEO, count:
- ☐ Clinic Administrator or CCO, count:
- ☐ Medical Director or CMO, count:
- ☐ Other staff not listed above, specify role & count:

Data Report Meetings

Since you received your last data report, did your program hold any meeting(s) to discuss the report? *

- (●) Yes
- ( ) No

reset

Please estimate the time spent at each meeting and indicate the role and count of staff who were involved below.

Activity	Time (in minutes)	Staff Role & Count
**Data report meeting 1**		☐ Peer Recovery Specialist, count:<br>☐ Substance Use Counselor, count:<br>☐ Mental Health Clinician, count:<br>☐ Nurse-RN/LPN, count:<br>☐ Clinical Supervisor, count:<br>☐ Prescriber - NP/PA, count:<br>☐ Prescriber - MD/DO , count:<br>☐ Program Director, count:<br>☐ Executive Director or CEO, count:<br>☐ Clinical Administrator or CCO, count:<br>☐ Medical Director or CMO, count:<br>☐ Other staff not listed above, specify role & count:
**Data report meeting 2**		☐ Peer Recovery Specialist, count:<br>☐ Substance Use Counselor, count:<br>☐ Mental Health Clinician, count:<br>☐ Nurse-RN/LPN, count:<br>☐ Clinical Supervisor, count:<br>☐ Prescriber - NP/PA, count:<br>☐ Prescriber - MD/DO , count:<br>☐ Program Director, count:<br>☐ Executive Director or CEO, count:<br>☐ Clinical Administrator or CCO, count:<br>☐ Medical Director or CMO, count:<br>☐ Other staff not listed above, specify role & count:
**Data report meeting 3**		☐ Peer Recovery Specialist, count:<br>☐ Substance Use Counselor, count:<br>☐ Mental Health Clinician, count:<br>☐ Nurse-RN/LPN, count:<br>☐ Clinical Supervisor, count:<br>☐ Prescriber - NP/PA, count:<br>☐ Prescriber - MD/DO , count:<br>☐ Program Director, count:<br>☐ Executive Director or CEO, count:<br>☐ Clinical Administrator or CCO, count:<br>☐ Medical Director or CMO, count:<br>☐ Other staff not listed above, specify role & count:

Additional Implementation Activities

Besides reviewing and discussing the last data report, has your program done any additional activities in the past quarter to prepare for the implementation of MOUD or improve access to MOUD within your program?

- (●) Yes
- ( ) No

Example are: setting up OUD screening procedures, developing staff training, establishing protocol for buprenorphine induction [illegible]

Example are: setting up OUD screening procedures, developing staff training, establishing protocol for buprenorphine induction. reset
*

Please list the activities, estimate the time spent, and indicate the role and count of staff who were involved below.

Activity	Time (in minutes)	Staff Role & Count
Expand		Peer Recovery Specialist, count:<br>Substance Use Counselor, count:<br>Mental Health Clinician, count:<br>Nurse-RN/LPN, count:<br>Clinical Supervisor, count:<br>Prescriber - NP/PA, count:<br>Prescriber - MD/DO , count:<br>Program Director, count:<br>Executive Director or CEO, count:<br>Clinical Administrator or CCO, count:<br>Medical Director or CMO, count:<br>Other staff not listed above, specify role & count:
Expand		Peer Recovery Specialist, count:<br>Substance Use Counselor, count:<br>Mental Health Clinician, count:<br>Nurse-RN/LPN, count:<br>Clinical Supervisor, count:<br>Prescriber - NP/PA, count:<br>Prescriber - MD/DO , count:<br>Program Director, count:<br>Executive Director or CEO, count:<br>Clinical Administrator or CCO, count:<br>Medical Director or CMO, count:<br>Other staff not listed above, specify role & count:
Expand		Peer Recovery Specialist, count:<br>Substance Use Counselor, count:<br>Mental Health Clinician, count:<br>Nurse-RN/LPN, count:<br>Clinical Supervisor, count:<br>Prescriber - NP/PA, count:<br>Prescriber - MD/DO , count:<br>Program Director, count:<br>Executive Director or CEO, count:<br>Clinical Administrator or CCO, count:<br>Medical Director or CMO, count:<br>Other staff not listed above, specify role & count:
Expand		Peer Recovery Specialist, count:<br>Substance Use Counselor, count:<br>Mental Health Clinician, count:<br>Nurse-RN/LPN, count:<br>Clinical Supervisor, count:<br>Prescriber - NP/PA, count:<br>Prescriber - MD/DO , count:<br>Program Director, count:<br>Executive Director or CEO, count:<br>Clinical Administrator or CCO, count:<br>Medical Director or CMO, count:<br>Other staff not listed above, specify role & count:
Expand		Peer Recovery Specialist, count:<br>Substance Use Counselor, count:<br>Mental Health Clinician, count:<br>Nurse-RN/LPN, count:<br>Clinical Supervisor, count:<br>Prescriber - NP/PA, count:<br>Prescriber - MD/DO , count:<br>Program Director, count:<br>Executive Director or CEO, count:<br>Clinical Administrator or CCO, count:<br>Medical Director or CMO, count:<br>Other staff not listed above, specify role & count:

Submit

Save & Return Later

Powered by REDCap - Cookie policy
